# Diverse Bacteriophages Infecting the Bacterial Striped Catfish Pathogen *Edwardsiella ictaluri*

**DOI:** 10.3390/microorganisms9091830

**Published:** 2021-08-28

**Authors:** Tan-Trung Nguyen, Tran T. T. Xuan, To H. Ngoc, Le T. My Duyen, Tu Q. Vinh, Pham D. T. My, Hoang A. Hoang, Le P. Nga

**Affiliations:** 1 Faculty of Chemical Engineering, Ho Chi Minh City University of Technology (HCMUT), 268 Ly Thuong Kiet, District 10, Ho Chi Minh City 700000, Vietnam; tan-trung.nguyen@hcmut.edu.vn (T.-T.N.); ttthanhxuan@hcmut.edu.vn (T.T.T.X.); 1612257@hcmut.edu.vn (T.H.N.); ltmduyen.sdh20@hcmut.edu.vn (L.T.M.D.); vinhbio1992@gmail.com (T.Q.V.); tramy191993@gmail.com (P.D.T.M.); hoang.a.hoang@hcmut.edu.vn (H.A.H.); 2 Vietnam National University Ho Chi Minh City, Linh Trung Ward, Thu Duc District, Ho Chi Minh City 700000, Vietnam

**Keywords:** bacteriophages, *Edwardsiella ictaluri*, *Edwardsiella* phage, genome comparison, genome analysis, *Autographiviridae*, *Myoviridae*

## Abstract

Bacteriophages infecting *Edwardsiella ictaluri* have been less investigated, although the host bacterium is one of the most important fish pathogens causing enteric septicemia of catfish (ESC). We present here two distinctly novel bacteriophages vB_EiM_PVN06 and vB_EiA_PVN09 infecting *Edwardsiella ictaluri* E1, with their geographical origins from the Mekong Delta, Vietnam. Bacteriophage vB_EiM_PVN06 native to a mud sample reveals complete differences of biological properties with the phage vB_EiA_PVN09 originated from a viscus of a healthy catfish (*Pangasianodon hypophthalmus*) cultured in the same area. Morphological analyses combined with genomic data indicate that phage vB_EiM_PVN06 is classified to *Myoviridae* family and shares high similarity with *E. ictaluri* phage PEi21 genome, while vB_EiA_PVN09 is a member of *Teseptimavirus* genus, *Autographiviridae* family, and mostly closes to phage vB_EcoP_IME390. The vB_EiA_PVN09 is a T7-like bacteriophage, which has been firstly found infecting to *E. ictaluri*, and host range analysis also evidences for the cross-infection of this phage to *Escherichia coli* K12 and *Escherichia coli* DH5α. Together, our research highlights the diversity of bacteriophages infecting the pathogen *E. ictaluri* and suggests further explorations of lytic phages in environmental niches, to be exploited in feasible strategies of phage therapy in ESC disease control.

## 1. Introduction

Enteric septicemia of catfish (ESC) with typical symptoms including white spots in the internal organs such as the liver, kidney, and spleen, caused by the primary fish bacterial pathogens *Edwardsiella ictaluri* and *Edwardsiella tarda*, was particularly recognized as a highly infectious disease of catfish families in different geographical locations in North America and Asia [1,2,3,4,5]. In Vietnam, *Edwardsiella ictaluri* was officially reported as a main causative agent of ECS in striped catfish *Pangasianodon hypophthalmus* and linked to disease outbreaks of catfish that caused a very high mortality rate (up to 90% in affected fishes) [2,6]. Remarkably, to treat and control the primary causative agent *E. ictaluri* in fish farms, abuse of antibiotics was highly prevalent; it caused negative impacts to the environment, antibiotic resistance, and over-accumulated antibiotic residues in frozen catfish products [7,8,9].

Bacteriophages, i.e., viruses infecting bacteria, that constitute 0.04% of the earth’s biomass were widely regarded to the largest and most diverse biological entities [10,11]. Due to the fascinating biological properties of phages, they were ideally considered to be employed as natural bio-control agents in alternative methods to fight against the pathogenic bacteria [12,13]. However, few studies about these natural predators of fish pathogen *E. ictaluri* were conducted [14,15,16]. To understand the diversity of phages infecting the enteric Gram-negative fish pathogen *E. ictaluri* and to identify lytic phages that could be exploited in effective bio-control strategies of the pathogen, we aimed to isolate, characterize, and sequence novel lytic *E. ictaluri* phages from different sources. In the current study, we reported the phenotypic properties and the complete nucleotide sequences of two novel bacteriophages specific to pathogen *E. ictaluri* isolated in Vietnam.

## 2. Materials and Methods

### 2.1. Phage Isolation and Purification

The bacterial strain *Edwardsiella ictaluri* E1 used as a host was isolated from the kidney and liver sample of diseased striped catfish in 2015 in Can Tho province, Mekong Delta, Vietnam. The strain at a dose of 10^5^ CFU.mL^−1^ caused the enteric septicemia of catfish (ESC) with the death toll by 50% (LD 50) for one hour of immersion. The host strain *E.* *ictaluri* E1 was verified by 16S rRNA gene sequencing and by PCR detection of representative genes of virulence-related pathogenicity islands such as *ureG* gene for bacterial adaptation in fish macrophage [17] and *evpC* gene for type VI secretion system [18]. The mud sample from a fish farm in Can Tho, Vietnam treated with 5% v/w chloroform, and then filtered with 0.22 μm membrane [19] was used to isolate the bacteriophage vB_EiM_PVN06 (PVN06). Bacteriophage vB_EiA_PVN09 (PVN09) was isolated from the homogenized internal organ of healthy fish grown in the same fish farm. Isolation, purification and storage protocols of the bacteriophage were described previously [20].

### 2.2. Host Range Analysis

The host range of phages was investigated by using various Gram-negative strains of bacteria (Table S1) in a drop plaque assay (spot test) on tryptic soy agar (TSA) plate [20].

### 2.3. Transmission Electron Microscopy (TEM)

Bacteriophage particles were prepared by negative staining with uranyl acetate 2% [21]. The morphologies of bacteriophages were observed by the transmission electron microscope (JEOL JEM-1010, Japan) at a voltage of 80 kV and an instrumental magnification of 25,000–30,000 at the National Institute of Hygiene and Epidemiology, Vietnam.

### 2.4. One-Step Growth Curve

One-step growth curve of phage was determined according to the method described previously [22] with some modifications [23]. Phage propagation parameters including burst size, latency period, and burst period were determined following the method described in [24].

### 2.5. Bacteriophage Stability at Acidic and Alkaline pH, in Solvent, and at Different Temperatures

PVN06 and PVN09 stocks prepared, respectively, at ~10^10^ PFU.ml^−1^ and ~10^9^ PFU.ml^−1^ were used as starting phage titer. For testing of phage stability in high temperature, the protocol was described by [22]. Phage stocks without dilution were incubated at 4, 15, 25, 30, 37, or 50 °C for 1 h. After 10 min, a sample was withdrawn and the active phage titer was measured by plaque assay. The protocol for testing phage stability in different pH was of [25]. Phage stock was diluted to ten times by fresh tryptic soy broth (TSB) adjusted with pH range of 3–11 using either 1M HCl or 1M NaOH. The diluted phage solutions were incubated at 30 °C for 24 h. Assays of phage stability in solvents were followed by method of [22,26]. Phage stock was diluted to two times by ethanol, ethyl acetate, chloroform, or dimethyl sulfoxide (DMSO) and mixed by vortexing. After one-hour incubation at 30 °C, the mixture was centrifuged and phage titer in the supernatant was measured by plaque assay. The survival phage titers in tested conditions were represented by the relative percentage of retained phage progenies.

### 2.6. Phage Genomic DNA Extraction, Sequencing and Bioinformatics Analysis

Prior to DNA extraction, purified phage stock solution was treated with DNase I and RNase at 37 °C for 1 h and then the enzymes were inactivated by heating at 85 °C for 15 min. Phage DNA genome was extracted by Phage DNA Isolation Kit (Norgen Biotek Inc., Thorold, ON, Canada) according to the manufacturer’s instruction. The purified bacteriophage genomes were examined with DNase I, RNase A (Thermo Fisher Scientific, Waltham, MA, USA), and Mung Bean nuclease (NEB, Ipswich, MA, USA) followed by the electrophoresis on 1% agarose gel to determine the kind of DNA genome. The bacteriophage genome was amplified by using the whole genome amplification (WGA) technique with EquiPhi29™ DNA Polymerase (Thermo Fisher Scientific). The WGA product was purified by the ethanol precipitation and then used as the DNA input for DNA library preparation by the Nextera XT DNA Library Prep kit, following the manufacturer’s instruction. The phage sequencing libraries were performed on the Illumina NextSeq550 sequencer with paired end, 150 bp per read at the Microbial Genome Sequencing Center (Pittsburgh, PA, USA). Whole-genome sequence data of phages PVN06 and PVN09 are available in the GenBank database under the following accession numbers MZ220765 (PVN06) and MZ220766 (PVN09).

Raw data were trimmed with Trimmomatic v0.39 using default parameters to remove adapter sequence [27]. The trimmed reads were then subsampled at one tenth by seqtk_sample (https://github.com/lh3/seqtk/, access on 20 August 2021). Subsampled reads were assembled with Unicycler configured with --mode, --min_fasta_length, -- linear_seqs, and --min_anchor_seg_len default [28]. Assembly errors were corrected with Pilon integrated in Unicycler by using default parameters [28]. The genome was annotated and determined ORFs using Prokka v1.14.6 with default parameters [29,30]. The potential functions of ORFs were predicted by using BLASTP on the non-redundant proteins database [31] and phmmer search on the UniProtKB database [32]. The large terminase (terL) amino-acid sequences from bacteriophage PVN06 and from the most similar phage were aligned with MAFTT [33] and phylogenetic trees were constructed by IQ-TREE using Maximum Likelihood method with 1000 bootstrap replicates [34]. A representative gene encoding the major capsid protein (MCP) was used for phylogenetic analysis of bacteriophage PVN09. The genomic comparisons between *Edwardsiella* phages and references were visualized by EasyFig tool [35].

## 3. Results

### 3.1. Two Novel Phages Infecting Edwardsiella Ictaluri Isolated in Vietnam Displayed Various Biological Features

**Figure 1:**
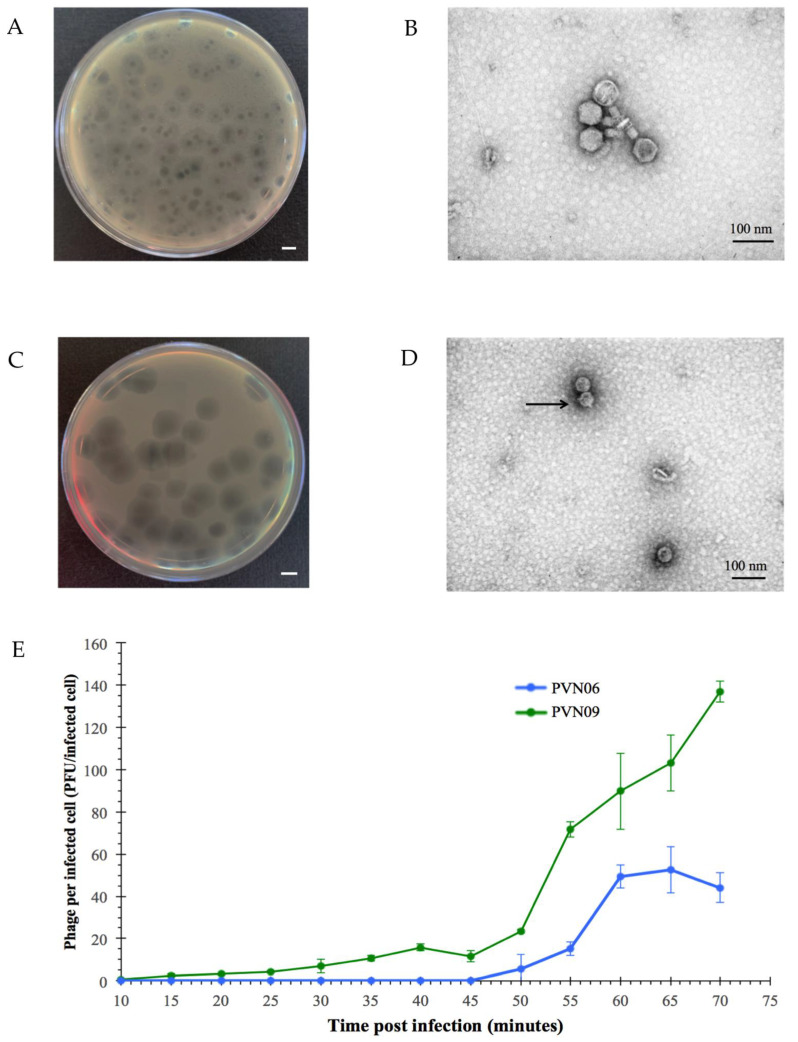
Characterization of *Edwardsiella ictaluri* phage PVN06 and PVN09. Top agar overlay showing plaque morphology of phage PVN06 (**A**) and PVN09 (**C**) on *E. ictaluri* E1. Scale bar indicates 1 cm. Transmission electron micrograph showing the tailed Myoviridae morphotype of PVN06 phage (**B**) and Podoviridae morphotype of PVN09 phage (**D**). The arrow indicates head structure of Podoviridae. (**E**) The one-step growth curves of the PVN06 phage (blue plot) and PVN09 phage (green plot) reveal progression of phage per infected cell over time with error bars showing standard deviation (SD). The values are means of three biologial repeats. The time period of 10–45 min was defined as eclipse period of PVN06 phage and the period of 10–50 min is for latency period of PVN09 phage. The burst period of PVN06 phage was defined as the time period of 45–70 min while the time interval of 50–70 min was defined for PVN09 phage.

*Edwardsiella ictaluri*, the pathogen causing enteric septicemia of catfish (ESC), was found among the most important fish diseases worldwide and emerged as an urgent threat to aquaculture. Bacteriophages were considered an effective method against the pathogen. However, only a few lytic phages infecting *E. ictaluri* have been isolated and investigated over the years [14,15,16]. As a strategy to identify novel phages that could be harnessed in phage therapy to control the pathogen in Mekong Delta, Vietnam, we isolated successfully two novel *E. ictaluri* phages (including vB_EiM_PVN06 (PVN06) and vB_EiA_PVN09 (PVN09)) from the mud sample and the homogenized internal organ of healthy fish, respectively. After purification, both phages showed a morphology of clear and circular plaque with constant diameter ~1.0 mm and ~3.0 mm, respectively, for PVN06 (Figure 1A) and for PVN09 (Figure 1C), surrounded by a translucent halo. The surrounding halo indicated a phage possibility in degradation of host extracellular polysaccharides [36]. To gain insight different plaque morphologies, we characterized further biological features of both phages. To determine the life cycle of new isolates on *E. ictaluri*, one-step growth assays were conducted. For phage PVN06, the latency period was found to be about 45 min and the burst period was about 60 min (Figure 1E). The PVN06 burst size was determined to be 52.5 ± 10.9 phage progenies per cell. The one-step growth curve of phage PVN09 showed a latency period of 50 min and a rise period of about 70 min, and the burst size was 137.1 ± 4.9 phage particles per infected cell (Figure 1E).

In order to determine the stability properties of *E. ictaluri* phages, we carried out assays of phage survival under various conditions of pH, temperatures, and solvents (Figure S1). Phage PVN06 exhibited its stability a wide range of pH (4–11) while PVN09 loss in viability in the narrow pH acidic range (4–6) and in the extremely alkaline pH (11). Thermal susceptibility test showed that the progenies retained of both phages remained inherently invariable from 4 °C to 37 °C. However, phage PVN09 had heat sensitivity at 50 °C. The phage PVN06 was found to be tolerance to chloroform and DMSO, in contrast to the susceptibilities of phage PVN09. The stabilities of two phages to external factors were consistent with features reviewed for *myovirus* and *podovirus* [37]. In addition, the various stability properties of these two phages in solvent were reflected through the condition of sample treatment during phage isolation and also their origin.

Our final purpose is the use of bacteriophage in bio-control strategy. Thus, the host range assay of the *E. ictaluri* phages was performed. The ability of host lysis in several common Gram-negative bacteria was tested by drop plaque assays (Table S1) and indicated that two novel phages were highly specific for bacterial hosts of *Edwardsiella* genus, *Edwardsiella ictaluri* and *Edwardsiella tarda*. Unexpectedly, phage PVN09 also caused strong lysis of *Escherichia coli* K12 and DH5α. Moreover, observing the one-step growth curve of phage PVN09 in host *E. coli* K12 showed a latency period of about 35 min and a stable phase obtained after approximately 65 min with an average burst size of 9.2 ± 1.9 phage particles per infected cell (Figure S2). In contrast to the host *E. coli* K12, the burst period of the PVN09 one-step growth curve in the host *E. ictaluri* was unstable and the plateau phase could not be obtained (Figure 1E). It most likely indicated that the cellular machinery of host *E. coli* might be more fitting for propagation of phage PVN09.

Due to the plentiful dissimilarity of plaque morphology, life cycle, phage stability, and host susceptibility of two *E. ictaluri* phages, we investigated more detailed the phage morphology by transmission electron microscopy (TEM). Indeed, two phages were different in morphology. Phage PVN06 exhibited an isometric polyhedral head that is approximately 54.9 ± 3.3 nm in diameter and a tail of approximately 54.7 ± 14.2 nm in length and 18.8 ± 3.3 nm in width (Figure 1B). The finding indicated that phage PVN06 belonged to the *Myoviridae* family. In contrast, TEM micrograph of PVN09 revealed the typical morphology of a *Podovirus* with an icosahedral head of 50.7 ± 2.9 nm in diameter. However, the tail was too short to be visible in TEM condition (Figure 1D). Phage PVN09 could be assigned to the *Autographivirinae* family in the *Caudovirales* order according to the current International Committee on Taxonomy of Viruses (ICTV) classification system. The morphology identification was also supported by the genomic data and BLASTn results of the phage genome. Altogether, these results indicated that the classification of two novel *E. ictaluri* phages in different phage families by TEM morphology was completely consistent with their various biological features and derivations.

### 3.2. Genomic Variations of Two Novel E. ictaluri Phages

**Figure 2:**
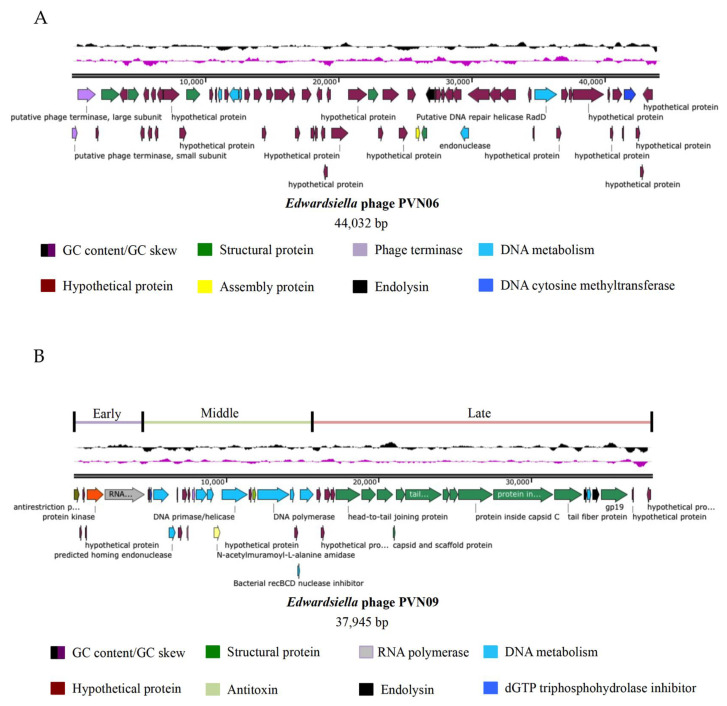
Linear genome map of the *E. ictaluri* phage PVN06 (**A**) and PVN09 (**B**). Annotations for predicted open reading frames (ORFs) are presented. The ORFs marks with different colors indicate categorized predicted functions of proteins. Two first plots represent a GC content (in black) and a GC skew (in violet) of the phage genome. *E. ictaluri* phage PVN09 is a T7-like phage and its genome can be divided into three parts of gene expression: early, middle, and late.

**Figure 3:**
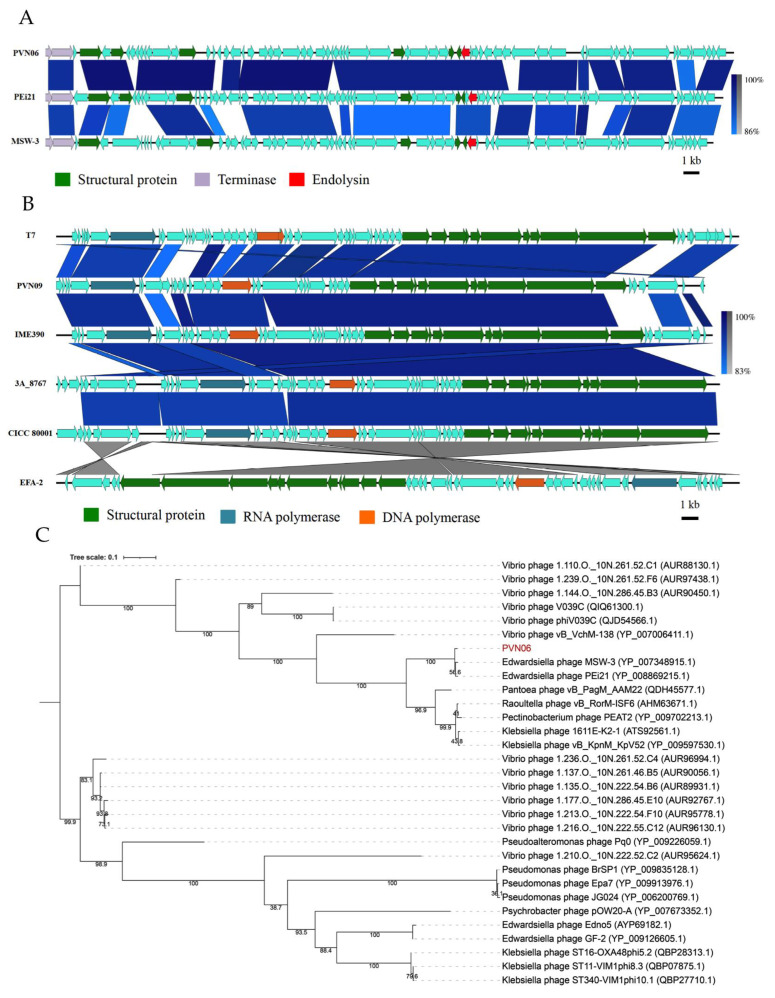

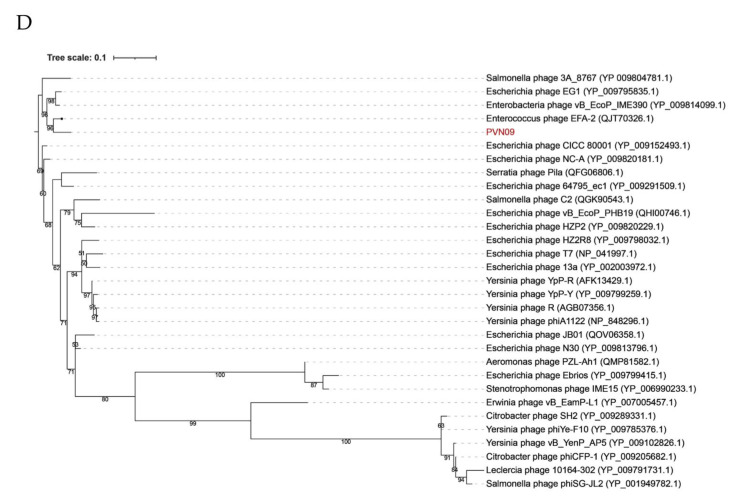
Genomic comparison of the *E. ictaluri* phage PVN06 and PVN09. (**A**) Schematic comparison of nucleotide sequences of phage PVN06 with sequences of *Edwardsiella* phage PEi21 (accession no. AP013057) and *Edwardsiella* phage MSW-3 (accession no. AB767244). (**B**) Genome comparison of phage PVN09 and selected representatives of phage T7 (accession no V01146), *Enterobacteria* phage vB_EcoP_IME390 (accession no. NC_048082), *Salmonella* phage 3A_8767 (accession no. MH382198), *Escherichia* phage CICC 80001 (accession no. NC_027387), *Enterococcus* phage EFA-2 (accession no. MT350293), using the Easyfig genome visualisation tool in BLASTn mode (version 2.2.3). Nucleotide similarity at different regions between genomes is given in percentages (right bar). The phage names are shortened and the functions of ORFs are marked with colors. Molecular phylogenetic analysis by the maximum likelihood method of BLASTp hits of large termirase sequences (TER) in phage PVN06 (**C**) or BLASTp hits of major capsid protein (MCP) in phage PVN09 (**D**). Sequences were aligned with MAFTT, and trees were constructed with IQ-TREE with 1000 bootstrap replicates. The percentage of trees in which the associated taxa clustered together is shown next to the branches (100 replicates). Each phylogenetic analysis was involved to 31 sequences.

Two novel *E. ictaluri* phages exhibited their distinct biological properties, so to get insight into their difference we determined their complete genome sequences. Assembly of PVN06 (average coverage X 326) revealed a 44,032 bp linear genome with a G + C content of 53% (Figure 2A and Table S2). The genomic size and the G + C content of PVN06 were equivalent to reported dwarf myoviruses on *Edwardsiella* genus as *Edwardsiella tarda* phage MSW-3 (42,746 bp, 53%) [15], *Edwardsiella ictaluri* phage PEi21 (43,378 bp, 52.6%) [16]. In addition, the G + C content of PVN06 was also quite similar to *E. ictaluri* (57.4%) strain isolated from other location [38] and thus shared the common trend in phage-host G + C relationships. Phage PVN06 harbored 69 ORFs (Figure 2A). Through protein database analysis, putative protein functions were assigned to 15 of the 69 ORFs (Figure 2A and Table S2). Moreover, PVN06 genome contained genes shared a similarity to counterparts found in two *Edwardsiella* dwarf myoviruses (Figure 3A and Table S3), associated with terminase, DNA metabolism, endolysin and structural proteins such as portal proteins, major capsid proteins, baseplate proteins, tail fiber assembly proteins, and inner membrane spanin subunit. Remarkably, the PVN06 genome did not own any gene encoding a protein product in DNA replication such as DNA polymerase, suggesting that the phage may employ host machinery to replicate its DNA genome. PVN06 also possesses a gene encoding cytosine-specific DNA methyltransferase which may be involved in DNA processing [39]. An overview of phage PVN06 genes annotated with predicted functions is provided in supplementary Table S3.

The genome of phage PVN09 (average coverage X 444) is a linear 37,945 bp in length with a G + C content of 49% (Figure 2B and Table S2). Interestingly, PVN09 genome has lower G + C content, in comparison with its host *Edwardsiella ictaluri* (G + C content of 57.4%) isolated from other location [38]. The genomic sequence of phage PVN09 is in contrast to the common trend of the reported *Myorividae* phages [15,16] or *Siphoviridae* phages [14], showing a similar G + C content to the hosts of *Edwardsiella* genus. Genome size of phage PVN09 also varies from these phages, consistently with data of its distinct phage morphology and biological features. Forty-eight putative open reading frames (ORFs) were identified in PVN09 genome. Putative protein functions were assigned to 31 of the 48 ORFs (Figure 2B and Table S2). An overview of PVN09 phage genes annotated with predicted functions is provided in supplementary Table S4. In phage PVN09, the genomic structure and modular organization of gene content consisting of gene clusters involved in host control, transcription, replication and regulation, morphogenesis, DNA packaging, and lysis were highly similar to those of typical T7-like phages (Figure 2B and Figure 3B). We identified eight strong T7 promoters, which were probably recognized by PVN09 RNA polymerase and served for high transcription of phage gene clusters during the lytic cycle. The PVN09 genome can be organized into three parts: early, middle, and late (Figure 2B and Table S4). The left end of PVN09 genome, corresponding to the early phase and likely transcribed by host RNA polymerase, harbored class I genes encoding an anti-restriction protein, a protein kinase inactivating host-catalyzed transcription, and a T7 RNA polymerase. The middle part consisted of class II genes that encoded mainly proteins involved in DNA metabolism, and included a host RNA polymerase inhibitor, a single-stranded DNA binding protein, an endonuclease, a primase/helicase, an inhibitor of toxin/antitoxin system, a DNA polymerase, a HNS-binding protein, an inhibitor of recBCD nuclease, and an exonuclease. The late region with class III genes encoded the virion structural proteins, as well as many of the proteins dedicated to maturation and cell lysis. A holin and an i-spanin that are required for host cell lysis and the process leading to release of virions, respectively, were also identified in the region. The genomic data indicated that PVN09 represented a clear case of typical T7-like phage, a member of *Autographivirinae* family, infecting the host *E. ictaluri*. The results of genome analysis of two *E. ictaluri* phages indicates that these phages were potentially lytic phages for phage therapy, since they did not contain any obvious antibiotic resistant gene, toxin gene, and lost the capacity for lysogeny or expression of virulence genes [40].

To further clarify the relationships of two *E. ictaluri* phages to other phages, genomic comparison and phylogenetic analysis were performed. A BLASTn search of whole PVN06 sequence genome resulted in two *Myoviridae* phage hits, for example as *Edwardsiella ictaluri* phage PEi21 (88% coverage, 97.65% sequence identity) and *Edwardsiella tarda* phage MSW-3 (79% coverage, 93.82% sequence identity). In addition, the pairwise sequence comparison (PASC) on whole sequence genome [41] indicated that the total nucleotide identity between PVN06 and PEi21 was 87.55%, or 78.34% for MSW-3 comparison. Genomes were then compared between PVN06, PEi21 and MSW-3 by using the Easyfig visualization tool in BLASTn mode (Figure 3A). As expected, the comparison result revealed highly similar synteny with two closely related *Myoviridae* phages. Based on the relatively conserved large terminase gene in the *Myoviridae* genomes, a maximum-likelihood phylogenetic tree was constructed (Figure 3C), with inclusion of the PEi21, MSW-3, and the other *Myoviridae* phages. The phylogenetic analysis placed PVN06 in a separate clade that was equivalent to another clade included two other members PEi21 and MSW-3. In agreement with the current guideline of the ICTV for species-level classification [42], and considering the differences in nucleotide (<95% nucleotide identity) between PVN06 and PEi21/MSW-3, it was proposed that phage PVN06 represents a separate and novel species within the *Yokohamavirus* genus, *Myoviridae* family which infected pathogen host *E. ictaluri*.

For phage PVN09, a Genbank BLASTn search revealed the genome hits of T7-like phages belonging to *Teseptimaviru*s genus, *Autographiviridae* family. Sequence alignment between PVN09 genome and the best hit of vB_EcoP_IME390 (accession no. NC_048082) in BLASTn produced a result of 91% coverage and 94% overall nucleotide identity. In support of this analysis, a genomic comparison between the phage PVN09 and four best genome hits of T7-like phages revealed a high similarity in gene synteny (Figure 3B). A maximum-likelihood phylogeny was reconstructed using the conserved major capsid protein (MCP) of T7-like phages, with the top 30 hits to the PVN09 MCP relied on Basic Local Alignment Search Tool Protein (BLASTP) analysis (Figure 3D). The phylogenetic analysis placed PVN09 in a separate clade including *Entorococcus* phage EFA-2 as the only other member, which was in a group including the clades with the T7-like phages as vB_EcoP_IME390 (accession no. NC_048082), EG1 (accession no. NC_047895), 3A_8767 (accession no. MH382198), members of the *Teseptimavirus* genus. The analyses were proposed that phage PVN09 represents a novel species of T7-like phage in *Teseptimavirus* genus.

## 4. Discussion

### 4.1. Various Approaches in Phage Isolation Revealed the Diversity of Edwardsiella Phages in Natural Habitat

Due to the potential utility of phages as bio-therapeutic agents against the infection of the striped catfish pathogen *E. ictaluri*, we commenced strategies to hunt lytic bacteriophages from different environment sources. The bacterial host *E. ictaluri*, a member of *Enterobacteriaceae* family, is abundant in water and can cause waterborne infection of catfish through invasion of oral route or gill [43]. Based on its habitat and infection pathways, we supposed that the natural sources with the host existence could be preferably used for phage isolation, for example as water samples, mud samples, and even the internal organs of striped catfishes in aquaculture farms. Reportedly, several bacteriophages against *E. ictaluri* were isolated using the water samples [14,16]. However, in our study, and unexpectedly, *E. ictaluri* phage was unsuccessfully isolated after labor-consuming screening of more than 300 aquaculture pond water samples collected from different positions of fish farms in the Mekong Delta, implying the extremely low abundance of phages specific to *E. ictaluri* in water. The mud samples, possibly favorite habitats of host *E. ictaluri*, were examined in an alternative approach of phage isolation. To reduce the sophisticated impacts of cohabited organisms during isolation process, mud samples were previously treated by chloroform. With this strategy, we obtained successfully a collection of lytic *Myoviridae* phages, in which vB_EiM_PVN06 is a representative. The phage PVN06 possessed a high similarity of myovirus morphology and genomic data as size, structure, and gene content (Figure 3A,B and Table S3) compared to *E. ictaluri* phage PEi21 isolated from Japanese river water [16], and consequently PVN06 was categorized as a new phage species to *Yokohamavirus* genus with only two members in the *Myoviridae* family. Phage PVN06 also shared high similarity of genomic sequence to a second member of the *Yokohamavirus* genus, *Edwardsiella tarda* phage MSW-3 [15] (79% coverage, 93.82% sequence identity) (Figure 3A,B and Table S3). The finding of phage PVN06 in the Mekong Delta, Vietnam implied the high similarity of host *E. ictaluri* using for phage isolation and the prevalence of members in *Yokohamavirus* genus specific to the bacterial genus *Edwardsiella* in different geographic locations in Asia. The genomic comparison of these phages, which showed the remarkable similarity to each other, suggested that they were likely members of a phage linage that was highly stable over time and in various geographic regions.

To raise the possibility of catching phage, the homogenized viscera of the healthy fishes were also paid attention to for phage isolation. In this approach, the internal-organ samples were untreated by chloroform to conserve the fish microbiota, and as expected, we successfully isolated the distinct novel phage vB_EiA_PVN09 varying greatly with phage PVN06 in biological features as plaque phenotype, growth curve, phage stability, and morphology, as well as in genome sequence. According to morphology and genome analysis of the phage PVN09, we firstly identified a T7-like phage classified to *Teseptimavirus* genus with diverse members, in *Autographiviridae* family, which was against the host *E. ictaluri*. In a similar approach, the virulent phage MK7 infected to *E. ictaluri* was also reported from an isolation source of kidney and liver of striped catfish. However, it was classified to *Myoviridae* family based on its morphological analysis [20]. Along with findings in last reports, the phages infecting to host *E. ictaluri* could span three different families including *Siphoviridae* [14], *Myoviridae* [16,20] and *Autographiviridae.* Finding phage PVN09 in internal organ of healthy striped catfish suggested that we should modify isolation approach of lytic *E. ictaluri* phages and investigate further the phage diversity in enteric system of catfish, as well as board host range phages.

### 4.2. A Probable Adaptation of Phage vB_EiA_PVN09 to the Host E. ictaluri

In relation to the source of phage isolation, it was not a surprise that phage vB_EiA_ PVN09 possessed the host cross-infection to *E. coli* K12 and DH5α. The isolation of PVN09 phage without chloroform treatment might convey unintentionally bacterial hosts of enteric system to the process, and consequently generating a condition of multiple host strains during isolation, which facilitated to catch board host range phages [44]. Indeed, the latency period of phage PVN09 in host *E. ictaluri* was more extended than in the bacterium *E. coli* K12. In addition, the one-step growth curve in *E. coli* K12 was more stable while the plateau phase in *E. ictaluri* could not be determined (Figure 1E and Figure S2). These results indicated the more effective growth of phage PVN09 in *E. coli* and the original host was seemingly *E. coli* existing in catfish. The survival of lytic phage in a new host primarily depended on their ability to entry the host, to overcome the host defenses, and to optimize the phage replication. It is likely that phage PVN09 passed virtually bacterial barriers and dominated successfully the host *E. ictaluri.*

## 5. Conclusions

Only four phages of *E. ictaluri* (mostly sampled in US and Japan) were previously analyzed at genomic level, but other geographic origins have been largely uninvestigated. To contribute to the understanding of *E. ictaluri* phage diversity, in this paper, we described two novel *E. ictaluri* phages isolated by different approaches in the Mekong Delta, Vietnam. The genomic analysis of two *E. ictaluri* phages supported the conclusion that these phages were potentially lytic phages for phage therapy. Our work expanded substantially the collection of the existing *E. ictaluri* phages and pointed in the genetic and phenotypic differences of phages, which were essential to bio-control strategies of bacterial pathogens based on phages in aquaculture.

## Data Availability

Data are contained within the article or supplementary data.

## Supplementary Materials

The following are available online at https://www.mdpi.com/article/10.3390/microorganisms9091830/s1. Figure S1: Biological stability of PVN06 phage and PVN09 phage under various conditions of treatment,; Figure S2: The one-step growth curve of the PVN09 phage on the host E. coli K12; Table S1: Host range testing of PVN06 and PVN09; Table S2: Summary of genomic features of PVN06 and PVN09; Table S3: Results of BLASTP, NCBI conversed domains and predictive ORFs of phage PVN06; Table S4: Results of BLASTP, NCBI conversed domains and predictive ORFs of phage PVN09; Table S5: Overview of best hits with phage PVN09 in a BLASTn search (pdf file).
