# Supplementary material for: Diverse Bacteriophages Infecting the Bacterial Striped Catfish Pathogen Edwardsiella ictaluri
Source: Microorganisms. 2021 Aug 28;9(9):1830. doi: 10.3390/microorganisms9091830 (PMC8465730; doi:10.3390/microorganisms9091830)
Supplement: Supplementary file 1 [file microorganisms-09-01830-s001.zip › microorganisms-1295286-supplementary.pdf]

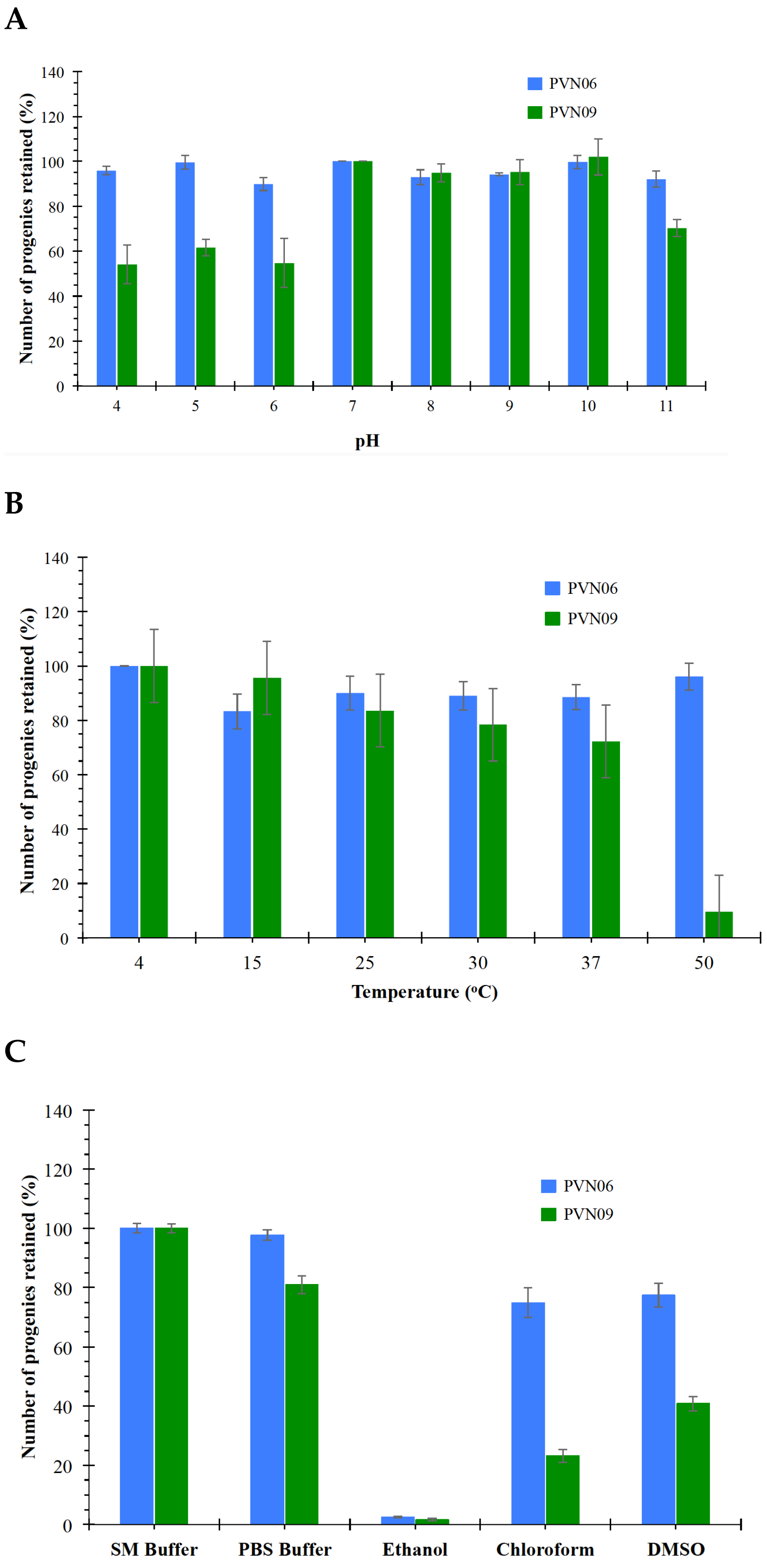

Figure S1. Biological stability of PVN06 phage (blue) and PVN09 phage (green) under various conditions of treatment as pH (A), temperature (B) and organic solvents (C) represented by decrease in progeny when comparing with the reference condition (100%) such as pH 7.0, 4°C and SM buffer, respectively. The shown values are means of three independent biological replicates with error bars indicating standard deviation (SD). DMSO is dimethyl sulfoxide.

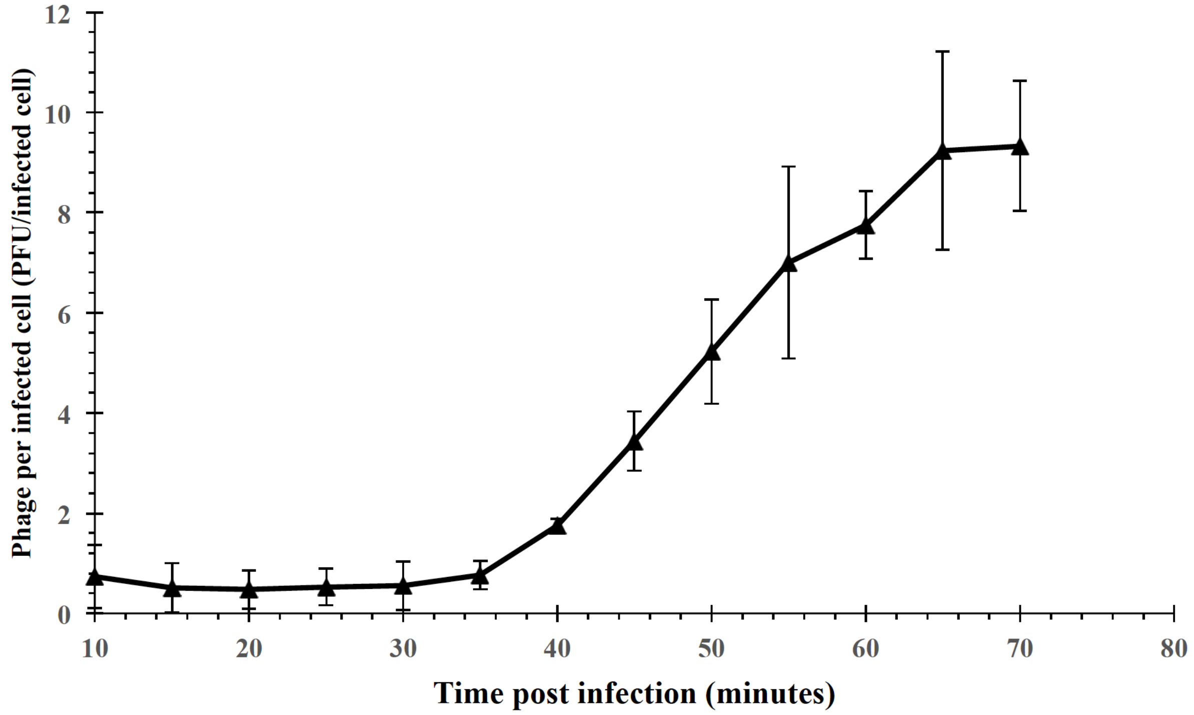

Figure S2. The one-step growth curve of the PVN09 phage on the host *E. coli* K12 shows progression of phage per infected cell over time with error bars showing standard deviation (SD). The values are means of three biological repeats. The time period 10 – 35 mins was defined as latency period of PVN09 phage. The busrt period of PVN09 phage was defined as the time period 35 – 65 mins.

Table S1. Host range testing of PVN06 and PVN09

Host Strain	Source	Susceptibility of phage	
		PVN06	PVN09
Edwardsiella ictaluri E1	CTU	++	+++
Edwardsiella tarda E2	CTU	++	++
Escherichia coli K12	ATCC	-	++
Escherichia coli DH5α	ATCC	-	+++
Aeromonas hydrophila A1	CTU	-	-
Aeromonas hydrophila A2	CTU	-	-
Entercocus faecalis ATCC 29212	ATCC	-	-
Staphylococus aureus ATCC 29213	ATCC	-	-
Staphylococus aureus ATCC 43300	ATCC	-	-
Pseudomonas aeruginosa ATCC 27853	ATCC	-	-
Salmonella enterotica ATCC 14028	ATCC	-	-

(+++) indicates bacterial strain is susceptible to phage infection, (++) indicates strain has an intermediate phenotype, and (-) indicates that the strain is resistant to phage infection. ATCC is abbreviated for American Type Culture Collection and CTU is for College of Aquaculture and Fisheries, Can Tho University, Vietnam.

Table S2. Summary of genomic features of PVN06 and PVN09

Phage	PVN06	PVN09
Genomic type	dsDNA	dsDNA
Genome length (bp)	44,032	37,945
G+C content (%)	53	49
Number of ORFs	69	48
Number of hypothetical proteins	54	17
Number of ORFs with predicted functions	15	31

Table S3. Results of BLASTP, NCBI conversed domains and predictive ORFs of phage PVN06

CDS	START (bp)	END (bp)	LENGTH (aa)	BLASTP				NCBI conserved domains		FINAL PREDICTED
				GENE PRODUCT	%Query Cover	E-value	% Match	DOMAIN	E-value	
orf1	1	420	139	putative terminase, small subunit [*Edwardsiella* phage MSW-3]	100%	1e-93	99%	Terminase_2 superfamily	1.38307e-05	Terminase small subunit
orf2	407	1795	462	phage terminase, large subunit [*Edwardsiella* phage PEi21]	100%	0.0	99%	Terminase_3 superfamily	1.37919e-36	Terminase large subunit
orf3	1792	2007	71	hypothetical protein G428_gp03 [*Edwardsiella* phage MSW-3]	69%	5e-25	94%	-	-	Hypothetical protein
orf4	2215	3588	457	putative portal protein [*Edwardsiella* phage PEi21]	99%	0.0	99%	DUF1073	2.04103e-107	Portal protein
orf5	3619	4149	176	hypothetical protein X848_gp05 [*Edwardsiella* phage PEi21]	100%	4e-80	44%	-	-	Hypothetical protein
orf6	4215	5024	269	putative head protein [*Edwardsiella* phage PEi21]	100%	0.0	98%	COG2369 superfamily	3.12602e-10	Head protein
orf7	5160	5387	75	hypothetical protein X848_gp07 [*Edwardsiella* phage PEi21]	91.18	6e-15	45%	-	-	Hypothetical protein
orf8	5384	5749	121	DUF551 domain-containing protein [*Edwardsiella*]	88	1e-38	73%	DUF551	3.12602e-10	Hypothetical protein
orf9	5746	5973	75	hypothetical protein [*Edwardsiella anguillarum*]	69.33	3e-26	100%	-	-	Hypothetical protein

orf10	5970	6254	94	hypothetical protein X848_gp08 [*Edwardsiella* phage PEi21]	94.68	2e-59	100%	-	-	Hypothetical protein
orf11	6257	6442	61	hypothetical protein X848_gp09 [*Edwardsiella* phage PEi21]	100	3e-37	100%	-	-	Hypothetical protein
orf12	6442	6816	124	hypothetical protein X848_gp10 [*Edwardsiella* phage PEi21]	100	7e-87	100%	-	-	Hypothetical protein
orf13	6863	8080	405	hypothetical protein X848_gp11 [*Edwardsiella* phage PEi21]	99.26	0.0	100%	DUF2213	6.51704e-62	Hypothetical protein
orf14	8080	8577	165	hypothetical protein X848_gp12 [*Edwardsiella* phage PEi21]	99.39	2e-110	100%	-	-	Hypothetical protein
orf15	8582	9646	354	putative major capsid protein [*Edwardsiella* phage MSW-3]	99.44	0.0	100%	DUF2184	3.08909e-47	Major capsid protein
orf16	10321	10590	89	hypothetical protein X848_gp16 [*Edwardsiella* phage PEi21]	98.88	2e-55	100%	-	-	Hypothetical protein
orf17	10728	10889	53	hypothetical protein X848_gp17 [*Edwardsiella* phage PEi21]	100	6e-26	92%	-	-	Hypothetical protein
orf18	10991	11230	79	phage antirepressor KilAC domain-containing protein [*Aeromonas jandaei*]	76.67%	5e-22	75%	Phage_pRha	1.11824e-14	Phage antirepressor KilAC domain-containing protein
orf19	11434	11781	115	hypothetical protein X848_gp19 [*Edwardsiella* phage PEi21]	100.00%	8e-77	100%	-	-	Hypothetical protein

orf20	11820	12485	221	putative DNA-binding protein [*Edwardsiella* phage PEi21]	99.10%	4e-161	100%	pRha superfamily	7.0209e-07	DNA-binding protein
orf21	12551	12706	51	DNA-binding protein [*Aeromonas caviae*]	81.82%	1e-15	86%	Phage_pRha superfamily	1.58389e-08	DNA-binding protein
orf22	12943	13365	140	hypothetical protein X848_gp39 [*Edwardsiella* phage PEi21]	99.35%	0.0	100%	DUF4054	7.59573e-08	Hypothetical protein
orf23	13662	14255	197	hypothetical protein X848_gp23 [*Edwardsiella* phage PEi21]	99.49%	1e-141	100%	-	-	Hypothetical protein
orf24	14257	14616	119	hypothetical protein X848_gp24 [*Edwardsiella* phage PEi21]	100%	4e-82	100%	-	-	Hypothetical protein
orf25	14606	15124	172	hypothetical protein X848_gp25 [*Edwardsiella* phage PEi21]	100%	6e-125	100%	-	-	Hypothetical protein
orf26	15189	16331	380	hypothetical protein X848_gp26 [*Edwardsiella* phage PEi21]	99.74%	0.0	100%	DUF3383 superfamily	5.39547e-22	Hypothetical protein
orf27	16345	16749	134	hypothetical protein X848_gp27 [*Edwardsiella* phage PEi21]	100%	2e-91	100%	-	-	Hypothetical protein
orf28	16749	17150	133	hypothetical protein X848_gp28 [*Edwardsiella* phage PEi21]	100%	5e-93	100%	-	-	Hypothetical protein
orf29	17299	17958	219	hypothetical protein G428_gp28 [*Edwardsiella* phage MSW-3]	100%	1e-156	100%	-	-	Hypothetical protein
orf30	17955	18251	99	hypothetical protein G428_gp29 [*Edwardsiella* phage MSW-3]	100%	6e-66	100%	-	-	Hypothetical protein

orf31	18252	18392	46	hypothetical protein X848_gp31 [*Edwardsiella* phage PEi21]	100%	1e-22	100%	-	-	Hypothetical protein
orf32	18379	18732	117	hypothetical protein X848_gp34 [*Edwardsiella* phage PEi21]	96.55%	8e-77	99%	-	-	Hypothetical protein
orf33	18729	18947	72	protein X848_gp35 [*Edwardsiella* phage PEi21]	100%	2e-45	100%	-	-	Hypothetical protein
orf34	18941	19141	66	hypothetical protein X848_gp36 [*Edwardsiella* phage PEi21]	98.48%	1e-39	100%	-	-	Hypothetical protein
orf35	19138	19401	87	hypothetical protein X848_gp37 [*Edwardsiella* phage PEi21]	98.85%	2e-58	100%	DUF2591 superfamily	1.60256e-06	Hypothetical protein
orf36	19461	20750	429	hypothetical protein X848_gp38 [*Edwardsiella* phage PEi21]	100%	0.0	100%	Lyz-like superfamily	0.00135438	Hypothetical protein
orf37	20747	22138	463	hypothetical protein X848_gp39 [*Edwardsiella* phage PEi21]	99.35%	0.0	100%	-	-	Hypothetical protein
orf38	22273	22998	241	putative baseplate protein [*Edwardsiella* phage PEi21]	97.93%	9e-170	100%	Phage_base_V superfamily	1.5658e-05	Baseplate protein
orf39	22995	23366	123	hypothetical protein X848_gp41 [*Edwardsiella* phage PEi21]	100%	7e-83	100%	-	-	Hypothetical protein
orf40	23338	24549	403	hypothetical protein X848_gp42 [*Edwardsiella* phage PEi21]	95.78%	0.0	100%	-	-	Hypothetical protein
orf41	24546	25190	214	hypothetical protein G428_gp40 [*Edwardsiella* phage MSW-3]	85.05%	7e-136	100%	DUF2612	1.01133e-17	Hypothetical protein

orf42	25192	25806	204	hypothetical protein X848_gp44 [*Edwardsiella* phage PEi21]	84.24%	1e-120	99%	-	-	Hypothetical protein
orf43	25806	26156	116	tail fiber assembly protein [*Edwardsiella* phage PEi21]	100%	9e-80	100%	Caudo_TAP superfamily	2.35753e-10	Tail fiber assembly protein
orf44	26291	26602	103	putative inner membrane spanin subunit [*Edwardsiella* phage PEi21]	100%	6e-65	100%	-	-	Inner membrane spanin subunit
orf45	26599	27132	177	putative SAR endolysin [*Edwardsiella* phage PEi21]	100%	2e-128	100%	endolysin_R21-like	1.61866e-44	SAR endolysin
orf46	27189	27314	41	hypothetical protein G428_gp45 [*Edwardsiella* phage MSW-3]	100%	4e-16	100%	-	-	Hypothetical protein
orf47	27317	27748	143	hypothetical protein X848_gp50 [*Edwardsiella* phage PEi21]	99.30%	1e-95	1e-95	-	-	Hypothetical protein
orf48	27760	28029	89	hypothetical protein G428_gp46 [*Edwardsiella* phage MSW-3]	98.88%	1e-54	100%	-	-	Hypothetical protein
orf49	28046	28573	175	hypothetical protein X848_gp52 [*Edwardsiella* phage PEi21]	86.25%	3e-45	45%	-	-	Hypothetical protein
orf50	28612	29208	198	hypothetical protein X848_gp54 [*Edwardsiella* phage PEi21]	98.97%	6e-138	98%	-	-	Hypothetical protein
orf51	29205	29753	182	HNH endonuclease [*Ochrobactrum* phage vB_OspM_OC]	37.50%	1e-32	89%	HNH_3	4.70991e-11	Endonuclease

orf52	29785	31314	509	hypothetical protein X848_gp54 [*Edwardsiella* phage PEi21]	80.75%	0.0	100%	-	-	Hypothetical protein
orf53	31362	32147	261	hypothetical protein X848_gp55 [*Edwardsiella* phage PEi21]	95.24%	0.0	100%	DUF2815	0.000998659	Hypothetical protein
orf54	32154	33305	383	hypothetical protein X848_gp56 [*Edwardsiella* phage PEi21]	99.48%	0.0	100%	Cas4_I-A_I-B_I-C_I-D_II-B superfamily	2.98807e-17	Hypothetical protein
orf55	34248	34454	68	hypothetical protein X848_gp58 [*Edwardsiella* phage PEi21]	95.52%	6e-27	98%	-	-	Hypothetical protein
orf56	34585	34752	10	-	-	-	-	-	-	Hypothetical protein
orf57	34746	36404	552	putative helicase [*Edwardsiella* phage PEi21]	97.46%	0.0	100%	SSL2	3.35686e-54	Helicase
orf58	36404	36748	114	hypothetical protein [*Salmonella* enterica]	61.06%	9e-45	99%	-	-	Hypothetical protein
orf59	36752	37252	166	hypothetical protein X848_gp62 [*Edwardsiella* phage PEi21]	98.80%	7e-117	100%	-	-	Hypothetical protein
orf60	37311	37595	94	hypothetical protein X848_gp63 [*Edwardsiella* phage PEi21]	98.94%	4e-62	100%	-	-	Hypothetical protein
orf61	37614	39962	782	hypothetical protein X848_gp64 [*Edwardsiella* phage PEi21]	99.62%	0.0	100%	COG4983 superfamily	5.19801e-23	Hypothetical protein
orf62	40213	40422	69	hypothetical protein G428_gp60 [*Edwardsiella* phage MSW-3]	93.75%	2e-37	92%	-	-	Hypothetical protein

orf63	40413	40631	72	hypothetical protein X848_gp66 [*Edwardsiella* phage PEi21]	100%	2e-45	100%	-	-	Hypothetical protein
orf64	40628	41311	227	hypothetical protein X848_gp67 [*Edwardsiella* phage PEi21]	81.50%	3e-117	100%	YjbI	6.53506e-39	Hypothetical protein
orf65	41308	41469	53	hypothetical protein G428_gp63 [*Edwardsiella* phage MSW-3]	98.11%	9e-29	100%	-	-	Hypothetical protein
orf66	41469	42341	290	DNA cytosine methyltransferase [*Edwardsiella tarda*]	77.33%	2e-163	98%	Cyt_C5_DNA_methylase superfamily	1.57858e-22	DNA cytosine methyl transferase
orf67	42341	42661	106	hypothetical protein G428_gp64 [*Edwardsiella* phage MSW-3]	95.28%	3e-70	100%	-	-	Hypothetical protein
orf68	42658	42939	93	hypothetical protein X848_gp70 [*Edwardsiella* phage PEi21]	100%	3e-61	100%	-	-	Hypothetical protein
orf69	42903	43547	214	hypothetical protein G428_gp66 [*Edwardsiella* phage MSW-3]	97.66%	2e-151	100%	PRK00247 superfamily	9.29183e-06	Hypothetical protein

Table S4. Results of BLASTP, NCBI conserved domains and predictive ORFs of phage PVN09

CDS	START (bp)	END (bp)	LENGTH (aa)	BLASTP				NCBI conserved domains		FINAL PREDICTED
				GENE PRODUCT	%Query Cover	E-value	% Match	DOMAIN	E-value	
orf1	1	366	121	putative antirestriction protein [*Shigella* phage VB_Ship_A7]	93.86%	2e-71	94%	ORC	3.20358e-54	Antirestriction protein
orf2	366	521	51	hypothetical protein CPT_Pila_005 [*Serratia* phage Pila]	94.12%	2e-27	100%	-	-	Hypothetical protein
orf3	555	716	53	hypothetical protein C2_008 [*Salmonella* phage C2]	88.46%	6e-22	98%	-	-	Hypothetical protein
orf4	710	847	45	hypothetical protein EFA2_00044 [*Enterococcus* phage EFA-2]	95.56%	2e-23	100%	-	-	Hypothetical protein
orf5	867	1946	359	protein kinase [*Escherichia* phage N30]	92.20%	0.0	100%	PHA00451	0	Protein Kinase
orf6	2019	4670	883	DNA-directed RNA polymerase [*Escherichia* phage CICC 80001]	97.85%	0.0	100%	PHA00452	0	DNA-directed RNA polymerase
orf7	4855	4983	42	hypothetical protein HOU37_gp06 [*Enterobacteria* phage vB_EcoP_IME390]	95.24%	1e-20	100%	PHA00453	3.95393e-12	Hypothetical protein

orf8	4985	5131	48	dGTP triphosphohydrolase inhibitor [*Escherichia* phage vB_EcoP_PHB19]	97.06%	4e-14	70%	DUF2745	1.62658e-12	dGTP triphosphohydrolase inhibitor
orf9	5229	6245	338	DNA ligase [*Escherichia* phage vB_EcoP_PHB19]	93.49%	0.0	100%	PHA00454	0	DNA ligase
orf10	6211	6672	153	predicted homing endonuclease [*Citrobacter* phage CR44b]	39.86%	3e-29	96%	HNH_3	8.54715e-14	Predicted homing endonuclease
orf11	6739	6828	29	hypothetical protein T7p12 [*Escherichia* phage T7]	82.76%	1e-07	100%	-	-	Hypothetical protein
orf12	6854	7114	86	hypothetical protein HOS93_gp05 [*Yersinia* phage YpP-Y]	96.51%	5e-51	100%	PHA00455	1.63594e-38	Hypothetical protein
orf13	7114	7428	104	hypothetical protein HOU37_gp11 [*Enterobacteria* phage vB_EcoP_IME390]	93.27%	5e-66	100%	DUF3310	2.44428e-19	Hypothetical protein
orf14	7418	7540	40	-	-	-	-	-	-	Hypothetical protein
orf15	7497	7646	49	hypothetical protein CKV1F_gp10 [*Escherichia* phage K1F]	91.67%	6e-25	97%	-	-	Hypothetical protein
orf16	7760	7954	64	Structure of T7 transcription factor Gp2-E. coli RNAp jaw domain complex [*Escherichia* phage T7]	96.88%	6e-39	100%	PHA00457	3.66917e-33	Structure of T7 transcription factor Gp2-E / Bacterial RNA polymerase inhibitor, chain A

orf17	8025	8729	234	ssDNA-binding protein [*Enterobacteria* phage vB_EcoP_IME390]	98.29%	3e-165	100%	PHA00458	4.69003e-127	ssDNA-binding protein
orf18	8730	9185	151	gp3 [Escherichia coli]	99.34%	7e-105	100%	PHA00159	8.81788e-102	gp3/Endonuclease
orf19	9185	9640	151	N-acetylmuramoyl-L-alanine amidase [*Enterobacteria* phage vB_EcoP_IME390]	98.68%	2e-107	100%	PHA00447	2.50503e-105	N-acetylmuramoyl-L-alanine amidase
orf20	9712	11415	567	DNA primase [*Enterococcus* phage EFA-2]	96.83%	0.0	100%	RecA-like_Gp4D_helicase	9.32722e-87	DNA primase
orf21	11487	11699	70	hypothetical protein T7p26 [*Escherichia* phage T7]	98.57%	2e-40	100%	DUF5471	1.41302e-29	Hypothetical protein
orf22	11719	11982	87	inhibitor of toxin/antitoxin system [*Escherichia* phage N30]	96.55%	1e-53	100%	TA_inhibitor superfamily	7.65567e-47	Inhibitor of toxin/antitoxin system
orf23	12063	14177	704	DNA-directed DNA polymerase [*Escherichia* phage HZ2R8]	97.59%	0.0	100%	DNA_pol_A superfamily	4.98608e-163	DNA polymerase
orf24	14197	14496	99	putative HNS binding protein [*Enterobacteria* phage vB_EcoP_IME390]	93.94%	2e-60	100%	DUF2675	7.52693e-57	HNS binding protein
orf25	14496	14705	69	hypothetical protein T7p31 [*Escherichia* phage T7]	98.55%	2e-42	100%	PHA00422	4.0644e-43	Hypothetical protein

orf26	14705	14863	52	inihibitor of recBCD nuclease [*Escherichia* phage CICC 80001]	94.23%	2e-27	100%	PHA00442	1.23828e-14	Bacterial recBCD nuclease inhibitor
orf27	14850	15752	300	exonuclease [*Enterobacteria* phage vB_EcoP_IME390]	97.67%	0.0	100%	PHA00439	0	Exonuclease
orf28	15951	16205	84	hypothetical protein HOT58_gp36 [*Salmonella* phage 3A_8767]	98.81%	2e-54	100%	PHA00438	1.33181e-49	Hypothetical protein
orf29	16210	16476	88	hypothetical protein C2_034 [*Salmonella* phage C2]	88.64%	1e-50	100%	PHA00441	2.2592e-32	Hypothetical protein
orf30	16476	16877	133	hypothetical protein T7p39 [*Escherichia* phage T7]	97.74%	8e-92	100%	-	-	Hypothetical protein
orf31	16881	17168	95	hypothetical protein HOT58_gp39 [*Salmonella* phage 3A_8767]	77.27%	9e-36	88%	VirionAssem_T7 superfamily	4.89158e-31	Hypothetical protein
orf32	17183	18793	536	head-to-tail joining protein [*Escherichia* phage CICC 80001]	99.25%	0.0	100%	Head-tail_con	9.50245e-114	Head-to-tail joining protein
orf33	18893	19816	307	capsid and scaffold protein [*Enterococcus* phage EFA-2]	96.42%	0.0	100%	PHA00435	6.26757e-159	Capsid and scaffold protein
orf34	19911	20942	343	capsid and scaffold protein [*Enterococcus* phage EFA-2]	96.50%	0.0	100%	PHA00201	0	Major capsid protein

orf35	20942	21100	52	capsid and scaffold protein [*Escherichia* phage NC-A]	90.38%	9e-20	100%	-	-	Capsid and scaffold protein
orf36	21165	21755	196	tail tubular protein A [*Escherichia* phage CICC 80001]	96.94%	4e-137	100%	PHA00428	3.09015e-102	Tail tubular protein A
orf37	21779	24163	793	tail tubular protein B [*Salmonella* phage 3A_8767]	94.45%	0.0	100%	-	-	Tail tubular protein B
orf38	24243	24659	138	protein inside capsid A [*Salmonella* phage 3A_8767]	97.83%	7e-98	100%	PHA00432	4.05297e-89	Protein inside capsid A
orf39	24663	25253	196	internal virion protein B [*Escherichia coli*]	93.37%	8e-129	100%	PHA00101	3.1004e-78	Internal virion protein B
orf40	25260	27503	747	protein inside capsid C [*Enterobacteria* phage vB_EcoP_IME390]	96.25%	0.0	100%	PHA00431	0	Protein inside capsid C
orf41	27530	31486	1318	protein inside capsid D [*Salmonella* phage 3A_8767]	97.34%	0.0	100%	PHA00368	0	protein inside capsid D
orf42	31559	33355	598	tail fiber protein [*Escherichia* phage Ebrios]	49.04%	2e-170	93%	Collar	5.52887e-06	Tail fiber protein
orf43	33504	33707	67	phage holin class II [*Stenotrophomonas* phage IME15]	100.00%	5e-39	100%	PHA00426	8.40258e-33	Phage holin class II
orf44	33713	33988	91	DNA packaging protein A [*Stenotrophomonas* phage IME15]	97.83%	3e-56	100%	DNA_Packaging_2 superfamily	1.09223e-45	DNA packaging protein A

orf45	34074	34511	145	i-spanin [*Escherichia* phage Ebrios]	95.17%	1e-97	100%	PHA00276	2.43304e-65	i-spanin
orf46	34613	36373	586	gp19 [*Escherichia* phage 13a]	95.73%	0.0	100%	termin_lrg_T7	0	gp19
orf47	36632	36781	49	gene 19.5 [*Escherichia* phage T7]	83.67%	7e-20	100%	PHA00406	8.85242e-09	Hypothetical protein
orf48	37649	37870	73	hypothetical protein CPT_Pila_003 [*Serratia* phage Pila]	74.19%	1e-20	83%	-	-	Hypothetical protein

Table S5. Overview of best hits with phage PVN09 in a BLASTn search

Name	Taxonomy	Host	Source	% cov.	% ident.	E-value	Accession
Escherichia phage vB_EcoP_PHB20	Unclassified	*Escherichia coli*	Sewage water	91%	94.01%	0.0	MN481366
Enterobacteria phage vB_EcoP_IME390	*Teseptimavirus*	*Escherichia coli* BL21	Sewage	91%	94.01%	0.0	NC_048082.1
Salmonella phage 3A_8767	*Teseptimavirus*	*Salmonella enterica subsp. enterica serovar Typhi* 8767	Wastewater from butcher house	92%	93.46%	0.0	NC_048004.1
Escherichia phage CICC 80001	*Teseptimavirus*	*Escherichia coli* HY05C	Unknown	92%	94.12%	0.0	KM242061.1
Escherichia phage 64795_ec1	*Teseptimavirus*	*Escherichia coli*	Unknown	91%	90.45%	0.0	KU927499.1
Escherichia phage vB_EcoP_PHB19	Unclassified	*Escherichia coli*	Sewage water	90%	89.86%	0.0	MN481365.1
Enterobacteria phage 13a	*Teseptimavirus*	*Escherichia coli*	Unknown	87%	92.12%	0.0	EU734174.1
Escherichia phage T7	*Teseptimavirus*	*Escherichia coli*	Unknown	92%	92.09%	0.0	LR745710.1
Enterobacteria phage T7 strain T7Del1revsplitRNAP	*Teseptimavirus*	*Escherichia coli*	Unknown	88%	92.09%	0.0	MG833025.1
Yersinia phage YpP-R	*Teseptimavirus*	*Yersinia pestis*	Unknown	88%	92.02%	0.0	JQ965701.1
Yersinia phage YpP-Y	*Teseptimavirus*	*Yersinia pestis*	Unknown	88%	91.98%	0.0	NC_047939.1
